# Access to Interdental Brushing in Periodontal Healthy Young Adults: A Cross-Sectional Study

**DOI:** 10.1371/journal.pone.0155467

**Published:** 2016-05-18

**Authors:** Florence Carrouel, Juan Carlos Llodra, Stéphane Viennot, Julie Santamaria, Manuel Bravo, Denis Bourgeois

**Affiliations:** 1 Institute of Functional Genomics of Lyon, University of Lyon, Lyon, France; 2 Department of Public Health, Faculty of Dental Medicine, University Lyon 1, Lyon, France; 3 Department of Public Health, Faculty of Dental Medicine, University of Granada, Granada, Spain; University of Brescia, ITALY

## Abstract

**Purpose:**

Interdental diameter space is largely undefined in adults, which compromises the decision support for daily interdental cleaning during routine practice in individual oral prophylaxis. This study assesses the distribution of diameter access of interdental spaces in an 18- to 25-year-old adult population free of periodontal disease.

**Methods:**

In March-April 2015, a cross-sectional study using random sampling was performed at the University Lyon 1, France. The interproximal dental spaces of 99 individuals were examined using a colorimetric calibrated probe associated with the corresponding calibrated interdental brush (IDB).

**Results:**

Of the 2,408 out of 2,608 sites, the overall accessibility prevalence of any interdental brushing was 92.3%. In total, 80.6% of the sites required interdental brushes with smaller diameters (0.6–0.7 mm). In anterior sites, the diameter of the interdental brushes used was smaller (55.8% of IDB with 0.6 mm) than the diameter of the interdental brushes used in posterior sites (26.1% of IDB with 0.6 mm) (p < 0.01). The adjusted ORs indicate a significant association with the location of the sites (approximately doubling the risk of bleeding, i.e., OR = 1.9, in posterior sites).

**Conclusions:**

Most interdental sites can be cleaned using interdental brushes. Even in healthy people, interdental hygiene requirements are very high. Strengthening the oral hygiene capacity by specifically using interdental brushes can have an effect on the health of the entire population. Screening of the accessibility of the interdental space should be a component of a routine examination for all patients.

## Introduction

Effective daily cleaning of interdental spaces is a challenge [1]. The removal of interproximal plaque to disrupt the biofilm is considered to be important for the maintenance of gingival health, prevention of periodontal disease and the reduction of caries [2]. Mechanical plaque control interrupts supragingival biofilm development, which enables the biofilm composition to be compatible with oral health in most patients [3, 4]. Biofilm colony detachment may result in the colonization of structures at a distant site, which may generate pathologies, such as bacterial endocarditis [5].

Conventional toothbrushing alone is not effective in removing biofilm between interdental spaces [6, 7]. Recommendations on oral hygiene practices from dental practitioners have largely focused on the methods of daily toothbrushing and interdental cleaning as standards to achieving and maintaining good oral health [6]. Dental floss is recommended for individuals with closed interdental spaces, and interproximal brushes are recommended for periodontal patients or in those with open embrasures [8, 9].

The interdental brush (IDB) currently represents the primary and most effective method available for interproximal cleaning compared with brushing alone or the combined use of tooth brushing and dental floss [10]. IDBs are specifically designed to clean between the teeth in accordance with the interdental space access diameter. The method of choice for interdental cleaning when brush space permits is to select the largest size that can penetrate into the interdental space and then to fill this space completely without causing discomfort.

No systematic reviews or publications have described the prevalence of the diameter of interdental spaces. Two authors (DB, JL) searched the following electronic databases: Embase via OVID (from 2000 to 20 December 2014), MEDLINE via OVID (1972 to 20 December 2014) and the Cochrane Library, but no studies were identified. Thus, this challenge is necessary to clarify the needs of healthy adults for efficient oral prophylaxis and for the control of dental plaque, periodontal diseases and dental caries. Thus, there is a need for a more comprehensive and rigorous assessment to ensure that the adult population is able to perform optimal biofilm disruption.

The purpose of this study was to provide estimates on the prevalence and extent of the diameter access of interdental spaces in the 18- to 25-year-old adult population free of periodontal disease. A second objective was to describe the environmental factors, which favor or limit the interdental diameter and thus the performance of daily IDB activities.

## Materials and Methods

### Ethics statement

The protocol was declared of public interest by the National Ethics Committee; was approved by the National Commission of Informatics and Liberties, France; and was performed in adherence to the Code of Ethics according to the Declaration of Helsinki. Written informed consent was obtained from all participants.

### Study design

This was an epidemiological analytical cross-sectional study.

### Study population

Recruitment and examination of the subjects was performed in March-April 2015 at the University School of Dental Medicine, Lyon, France. The study population consisted of community students, both males and females, between 18 to 25 years old. The participants were randomly recruited in 2014 through a register of 590 subjects. Stratification by gender occurred at the enrolment step. The decision to participate was on a voluntary basis. The final study sample of interdental sites (30 interdental sites/subject) with sufficient space for the IDB consisted of 2408 sites. After considering a design effect (i.e., the interproximal sites are clustered within the patients) of 5.0 (estimated from the first 20 subjects studied) in estimating the accessibility of the interdental percentage, this sample size was larger than that required (n = 384 x 5.0 = 1920) to estimate the proportion of interdental access to an accuracy of 5%, considering a priori the worst of possibilities (p = 0.5). The minimum sample population required to comply with these criteria was estimated as 99 adults. In total, 100% of the students agreed to participate in the survey.

Subjects were included if they 1) were 18–25 years of age; 2) had at least 20 natural scorable teeth, including 3^rd^ molars; 3) had no signs of clinical periodontitis (distance from free gingival margin to the bottom of the sulcus or periodontal pocket ≤ 3 mm); 4) had no significant dental anomalies or prosthetic restorations or interproximal caries; 5) had declared at least two tooth brushings per day and 6) had no health condition that required antibiotic prophylaxis before interproximal probing.

Subjects were excluded from the study if they had 1) systemic or oral antibiotic treatment at any time during the previous 3 months; 2) significant oral care or orthodontic or professional prophylaxis within the preceding 4 weeks; 3) evidence of any other concomitant systemic disorders, or diseases affecting the immune system; 4) medication such as anti-platelet or anti-coagulant agents and 5) a history of periodontal care.

### Internal Validity

**Fig 1:**
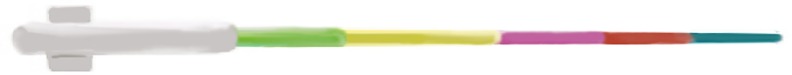
IAP CURAPROX probe.

Both experienced dentists with graduate training in periodontics were trained in the use of the IAP CURAPROX probe (Curaden International AG, Kriens, Switzerland) (Fig 1). The pressure applied by a horizontal probe in the interdental area should be firm and continuous until reaching maximum compression with minimal discomfort to the patient. A Visual Analogical Scale (VAS) was used to estimate the patient’s discomfort perception correlated to interproximal pressure (0 = no pain, 10 = unbearable pain) [11].The force ranged between 100 and 200 cN (0.40–0.80 gram force), and 80% of subjects assigned a VAS score ≤ 3 to their discomfort. From this observation, it can be assumed that a correct IDB classification recording force should induce minimal patient discomfort. Examiners were blinded to each other, and the two observations were taken at an interval of at least 15 minutes. The kappa statistic was 0.76 (95% CI: 0.14–1.38; p = 0.02), indicating excellent interoperator agreement according to Landis and Koch [12].

### Protocol

The clinical trial consisted of the following two steps: 1) a screening examination to identify the eligibility of the subjects and 2) an interproximal baseline examination. During the screening examination, subjects were screened for suitability by a periodontist, and consent was obtained. At the second visit, a colorimetric probe evaluated all of the interproximal spaces and then the corresponding interdental brush (Curaprox CPS, Curaden International AG, Kriens, Switzerland) was introduced. No subject was identified as having a periodontal disease at step 1.

### Interproximal Clinical Examination

All of the interproximal spaces were evaluated using the IAP colorimetric probe. The IAP CURAPROX calibrating probe is a graduated conical instrument with a rounded tip. The working part consists of colored bands from the point to the base, corresponding to IDBs by increasing diameter. The largest section of each colored band corresponds to the cleaning efficiency diameter of the relevant brush. The non-working part has a click-fastening joint for the attachment of a handle for easier use and access to the interproximal spaces at the back of the mouth.

The procedure consists of horizontally introducing the IAP CURAPROX probe into the vestibular interdental space, inserting it fully, and then noting the color emerging from the interdental space on the vestibular side [13]. This color corresponds to the color code of the IDB that is most suitable for the space calibration in question. Next, the corresponding brush was introduced into the interproximal space. Thirty potential registered sites/subjects were notified.

### Scoring Method

#### Classification of Access Diameter for Interdental Space

The IDBs used are from the CPS range of CURAPROX (Table 1). This pack consists of 5 cylindrical IDBs with the following characteristics:

- a color code related to the size of the brush;
- an access diameter, defined by the gauge of the CURAL wire core as “1 –Blue, 2 –Red, 3 –Pink, 4 –Yellow and 5 –Green,” was used to stiffen the IDB; and
- an effective cleaning diameter defined by the length of the synthetic bristles covering the working part of the device.

**Table 1:** Characteristics of the IDBs in relationship to the classification of the access diameter for the interdental space.

Color code	Blue (B)	Red (R)	Pink (P)	Yellow (Y)	Green (G)
Access diameter (mm)	0.6	0.7	0.8	0.9	1.1
Effective cleaning diameter (mm)	2.2	2.5	3.2	4.0	5.0

#### Secondary outcomes

Analyses were defined for variables that could affect the interdental diameter.

The healthy bleeding gingival site was recorded (i.e., yes or no). A site was defined as healthy (i.e., yes) if it was assigned either Code 0, indicating healthy gingival tissues with no bleeding, or Code 1, indicating that bleeding representing tissue reaction to horizontal pressure in the interdental area applied by an interdental brush was present. Bleeding is scored as either present or absent for each interdental site after 30 s. Interdental brushes can be considered a valid alternative to a periodontal probe in assessing marginal bleeding in gingivitis patients [14]. Subject defined baseline bleeding risk according to the percentage of bleeding interdental sites was determined to be high-level if the subject had bleeding sites ≥ 30% and low-level if the subject had bleeding sites < 30%.

Tobacco use, age, and gender were also recorded.

### Analysis

The statistical unit was the interdental space. The analysis was performed at a global level, including all interdental sites, and considering the five categories of interdental score as an ordinal scale, from 1 to 5. Furthermore, the analysis was performed according to the location of the sites, which included anterior (up to distal of second incisor) and posterior (from distal of canine to distal of second molar). At the site level, the analysis was performed with SUDAAN 7.0 (RTI, RTP, NC) to account for clustering (multiple sites within the mouth) in p-value and standard errors calculations. At the patient level, we used SPSS 15.0 (SPSS Inc., Chicago, IL).

## Results

**Fig 2:**
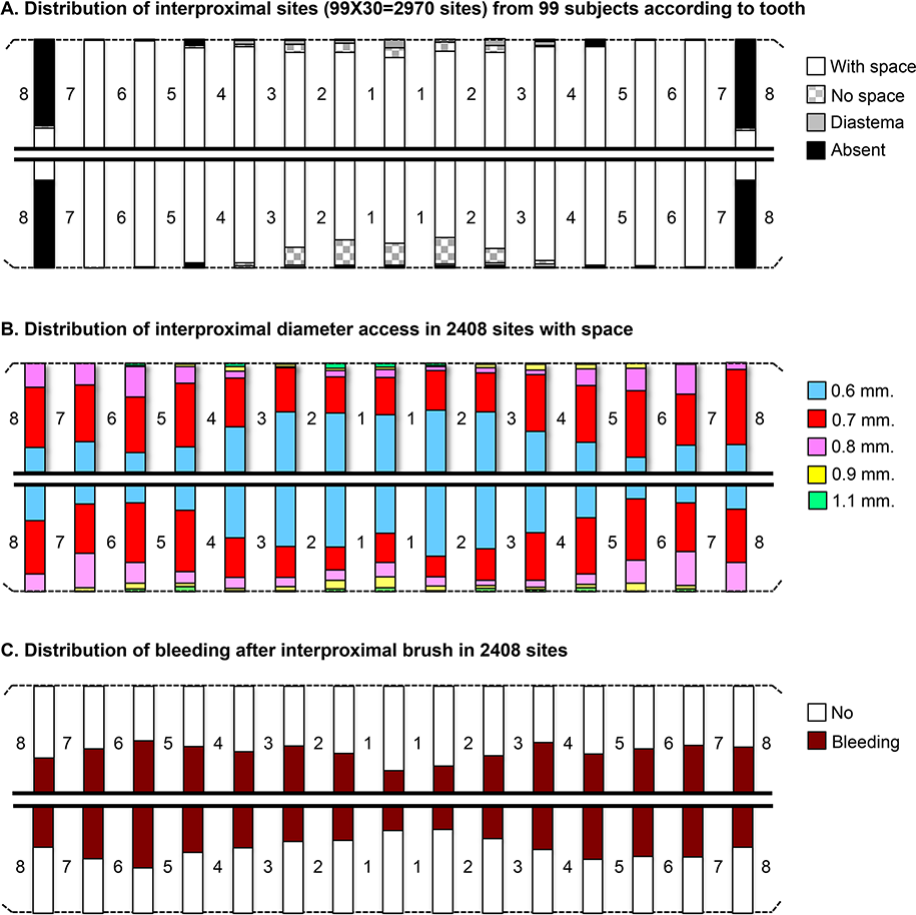
Distribution of characteristics in interproximal sites (30/subject), denoted in bars, corresponding to 99 adults*. (A) Distribution of interproximal sites (99 x 30 = 2970 sites) from 99 adults according to the type of tooth. (B) Distribution of interproximal diameter access in 2408 sites with spaces. (C) Distribution of bleeding after interproximal brush in 2408 sites. * Note that the teeth are numbered.

The sample consisted of 99 subjects (44 females and 24 smokers) with a mean age of 22 ± 2.7 years. From 2970 (99 subjects x 30 sites/subjects) potentially eligible sites, 2608 sites remained after the exclusion of an absence of teeth (362). One-hundred and fifty-three interproximal spaces were too small to introduce IDB, and 47 presented diastema. Thus, in the study, 2408 out of 2608 sites could be used for IDB (92.3%) (Fig 2A). The distribution of the diameter of interdental brushes with anterior and posterior site values is reported in Fig 2B. Finally, a higher prevalence of bleeding in posterior sites compared to anterior sites is shown in Fig 2C.

Among the 30 potential registered sites/subjects, 1.5 sites did not have sufficient space to introduce the IDB, 0.5 for diastema and 3.7 with no teeth. Thus, 24.3 average adult interdental sites demonstrated sufficient space to allow access to IDB. Moreover, 8.78 ± 5.18 sites were classified as grade 1 vs. 10.83 ± 4.56 as grade 2 and 3.39 ± 3.47 as grade 3. The scores 4 (0.83 ± 1.42) and 5 (0.49 ± 1.66) were very low. Only 0.60% of adults had ≥ 30% bleeding sites. The distribution of diameter severity for the interdental space by subject was not affected by gender, tobacco use or the adult’s baseline bleeding (Table 2).

**Table 2:** Distribution (mean ± sd) of the number of interproximal sites according to the diameter of the interproximal brush in 99 adults.

		IDB diameter (mm.)				
	n	0.6	0.7	0.8	0.9	1.1
All	99	8.78 ± 5.18	10.83 ± 4.56	3.39 ± 3.47	0.83 ± 1.42	0.49 ± 1.66
Sex						
Male	55	8.27 ± 5.07	10.80 ± 4.50	3.74 ± 3.52	0.98 ± 1.51	0.60 ± 2.01
Female	44	9.41 ± 5.31	10.86 ± 4.68	2.95 ± 3.38	0.64 ± 1.30	0.36 ± 1.06
p-value		0.280	0.944	0.260	0.229	0.510
Smoker						
No	75	9.03 ± 5.29	10.83 ± 4.35	3.11 ± 3.15	0.79 ± 1.33	0.37 ± 0.98
Yes	24	8.00 ± 4.86	10.83 ± 5.26	4.29 ± 4.27	0.96 ± 1.71	0.87 ± 2.89
p-value		0.594	0.991	0.142	0.615	0.195
Subject's Baseline Bleeding						
Low (< 30% bleeding sites)	39	7.85 ± 4.74	10.74 ± 4.87	4.18 ± 3.82	1.10 ± 1.82	0.67 ± 1.32
High (≥ 30% bleeding sites)	60	9.38 ± 5.40	10.88 ± 4.39	2.88 ± 3.14	0.65 ± 1.07	0.38 ± 1.84
p-value		0.147	0.877	0.065	0.118	0.587
		**IDB diameter (mm.)**				
	n	0.6	0.7	0.8	0.9	1.1
**All**	99	8.78 ± 5.18	10.83 ± 4.56	3.39 ± 3.47	0.83 ± 1.42	0.49 ± 1.66
**Sex**						
Male	55	8.27 ± 5.07	10.80 ± 4.50	3.74 ± 3.52	0.98 ± 1.51	0.60 ± 2.01
Female	44	9.41 ± 5.31	10.86 ± 4.68	2.95 ± 3.38	0.64 ± 1.30	0.36 ± 1.06
p-value		0.280	0.944	0.260	0.229	0.510
**Smoker**						
No	75	9.03 ± 5.29	10.83 ± 4.35	3.11 ± 3.15	0.79 ± 1.33	0.37 ± 0.98
Yes	24	8.00 ± 4.86	10.83 ± 5.26	4.29 ± 4.27	0.96 ± 1.71	0.87 ± 2.89
p-value		0.594	0.991	0.142	0.615	0.195
**Subject's Baseline Bleeding**						
Low (< 30% bleeding sites)	39	7.85 ± 4.74	10.74 ± 4.87	4.18 ± 3.82	1.10 ± 1.82	0.67 ± 1.32
High (≥ 30% bleeding sites)	60	9.38 ± 5.40	10.88 ± 4.39	2.88 ± 3.14	0.65 ± 1.07	0.38 ± 1.84
p-value		0.147	0.877	0.065	0.118	0.587

Among the registered sites, 40.0% (95% CI: 0.35–0.45) showed bleeding upon IDB passage. Globally, 80.6% of the sites required interdental brushes with a smaller diameter (diameter of 0.6–0.7 mm). In the anterior location, a 0.6-mm brush was needed in approximately 56% of the sites, resulting in it becoming the most commonly used brush. In the posterior location, a 0.7-mm brush was required in approximately 51% of the sites. The distribution of the interdental brushes in the anterior and posterior sites was significantly different (p < 0.001). The prevalence of bleeding is higher in posterior than in anterior sites, globally and for the majority of brush sizes, as shown in Table 3, where the 95%-CIs do not overlap. Furthermore, an inverse relationship between brush diameter and bleeding prevalence was found.

**Table 3:** IDB and bleeding in sites with spaces (n = 2408) in 99 adults.

	All (n = 2408)		Anterior (n = 812)		Posterior (n = 1596)	
	Brush^a^	Bleeding	Brush	Bleeding	Brush	Bleeding
IDB	n (%)	% (95%-CI)^b^	n (%)	% (95%-CI)	n (%)	% (95%-CI)
**All**	2408 (100)	40 (35–45)	812 (100)	31 (26–37)	1596 (100)	45 (39–50)
**IDB diameter**						
1 (0.6 mm.)	869 (36.1)	41 (35–47)	453 (55.8)	36 (29–43)	416 (26.1)	46 (39–54)
2 (0.7 mm.)	1072 (44.5)	43 (36–49)	253 (31.2)	31 (24–39)	819 (51.3)	46 (39–53)
3 (0.8 mm.)	336 (14.0)	39 (31–48)	53 (6.5)	15 (4–26)	283 (17.7)	44 (35–53)
4 (0.9 mm.)	82 (3.4)	24 (12–37)	32 (3.9)	6 (0–15)	50 (3.1)	36 (20–52)
5 (1.1 mm.)	49 (2.0)	14 (4–24)	21 (2.6)	5 (0–11)	28 (1.8)	21 (5–38)

a The comparison of brush distribution between anterior and posterior teeth is p < 0.001 with CROSSTAB procedure in SUDAAN.

b 95%-Confidence Intervals corrected for complex sampling (multiple sites within the mouth), with the DESCRIPT procedure in SUDAAN.

Table 4 shows the distribution of the different interdental brushes according to the principal studied variables. Gender (p = 0.16) and tobacco (p = 0.20) have no effect on the distribution of the interproximal brush diameter. However, a lower prevalence of larger brush diameters was observed at the sites of subjects classified as having a high bleeding risk (p = 0.06).

**Table 4:** Distribution of interproximal sites (n = 2408^a^) from 99 adults according to adult’s variables.

		Brush diameter (mm.) (%↔)					
	n	0.6	0.7	0.8	0.9	1.1	p-value^b^
Sex							0.161
Female	1066	38.8	44.8	12.2	2.6	1.5	
Male	1342	33.9	44.3	15.4	4.0	2.5	
Smoker							0.205
Yes	599	32.1	43.4	17.2	3.8	3.5	
No	1809	37.4	44.9	12.9	3.3	1.5	
Patient's periodontal risk							0.061
Low (< 30% bleeding sites)	957	32.0	43.8	17.0	4.5	2.7	
High (≥ 30% bleeding sites)	1451	38.8	45.0	11.9	2.7	1.6	
		**Brush diameter (mm.) (%↔)**					
	n	0.6	0.7	0.8	0.9	1.1	p-value^b^
**Sex**							0.161
Female	1066	38.8	44.8	12.2	2.6	1.5	
Male	1342	33.9	44.3	15.4	4.0	2.5	
**Smoker**							0.205
Yes	599	32.1	43.4	17.2	3.8	3.5	
No	1809	37.4	44.9	12.9	3.3	1.5	
**Patient's periodontal risk**							0.061
Low (< 30% bleeding sites)	957	32.0	43.8	17.0	4.5	2.7	
High (≥ 30% bleeding sites)	1451	38.8	45.0	11.9	2.7	1.6	

a Interproximal sites with enough space to introduce the IDB (interdental brush).

b DESCRIPT procedure in SUDAAN 7.0 used to adjust for clustering (multiple sites within the patient).

Table 5 presents, as a secondary outcome, the univariate and multivariate associations of the studied variables with interproximal bleeding after IDB as a dependent variable. Crude (univariate effects) and adjusted (multivariate effects) ORs were very similar, indicating that confounding effects are low in this sample, at least for the studied variables. The adjusted ORs indicate a significant association with the zone–an approximate doubling of the risk of bleeding, i.e., an OR = 1.9, in posterior sites–and with IDB diameter–an inverse relationship between diameter and bleeding–. Gender and tobacco were not significantly associated with bleeding.

**Table 5:** Univariate and multivariate associations^a^ between studied variables and interproximal bleeding after IDB (n = 2408 sites).

		Bleeding		Univariate	Multivariate
Variable	n	%	(95%-CI)	OR^b^ (95%-CI)	OR (95%-CI)
**Sex**				p = 0.252	p = 0.313
Female	1066	44	(36–51)	1.3 (0.8–1.9)	1.2 (0.8–1.9)
Male	1342	38	(31–44)	1.0	1.0
**Smoker**				p = 0.769	p = 0.870
Yes	599	39	(31–47)	0.9 (0.6–1.4)	1.0 (0.6–1.5)
No	1809	41	(35–47)	1.0	1.0
**Zone**				p < 0.001	p = < 0.001
Posterior	1596	45	(39–51)	1.8 (1.4–2.3)	1.9 (1.5–2.4)
Anterior	812	31	(26–37)	1.0	1.0
**IDB diameter**				p = 0.002	p = 0.003
1 (0.6 mm.)	869	41	(35–47)	4.2 (1.8–9.5)	4.4 (1.9–10.0)
2 (0.7 mm.)	1072	43	(36–49)	4.4 (1.9–10.2)	3.9 (1.7–9.0)
3 (0.8 mm.)	336	39	(31–48)	3.9 (1.8–8.5)	3.3 (1.5–7.2)
4 (0.9 mm.)	82	24	(12–37)	1.9 (0.7–5.4)	1.9 (0.7–5.1)
5 (1.1 mm.)	49	14	(5–24)	1.0	1.0

a Univariate and multivariate (forcing all variables) associations with p-values and 95% CI estimated with LOGISTIC PROC in SUDAAN 7.0, to account for clustering (multiple sites -408- within patients -99-).

b Odds ratio

## Discussion

### Summary of evidence

To the best of our knowledge, the present study is the first cross-sectional study to describe the prevalence of access to interdental brushing in adults in a healthy periodontal condition. The results pertaining to a random sample of 18- to 25-year-old students suggest that 100% of these students have access to IDBs. In addition, the number of sites identified per individual is high with the majority indicating brushes of a small diameter (0.6–0.7 mm). IDBs from the CPS range of CURAPROX could penetrate into 94% of interdental spaces. Our student population demonstrated a high potential of individual oral prophylaxis according to interproximal brush diameter in a study group that was considered to be a very low risk model for periodontal diseases. Taken together, these results demonstrated substantially unmet IDB needs for the interproximal sites of French young adults.

Fundamentally, the use of interproximal brushes remains an important oral hygiene problem among individuals, affecting 43% of those admitted. No other reasons, except medical considerations, would limit the use of IDB in healthy subjects. Dentists should choose IDB based on the patient’s clinical situation and the scientific evidence supporting their usage and devices with alternative dimensions that can be used in specific situations.

Our results may underestimate the burden of needs. Subjects with more severe diseases, such as the concept of multimorbidity, which refers to the co-occurrence of multiple diseases or medical conditions within one person and its negative health consequences, were not included. However, we were examining a highly educated sample, in which oral hygiene was considered positive to the extent that they declared they brushed their teeth at least twice daily.

It is difficult to categorize the interdental spaces in an individual as normal, below normal or above normal on the basis of a single parameter. Moreover, the differences in the values of interdental spaces in different sites could be extreme. Few studies have been performed in order to identify the factors that result in the formation or incomplete formation of the diameter of space between natural and permanent teeth. Our study was not designed to characterize the relationship between interdental space and the identification of factors, nor was it developed to evaluate the effect of ID access on the risk of periodontal disease. Bleeding, which reflects the level of hygiene of the interdental spaces [15] and tobacco use prevalence were nonetheless identified.

An unexpected finding was the generally high prevalence of tobacco use (39.0%) and the high rate of adults with bleeding sites ≥ 30% (69.9%), which do not have a significant effect on the diameter of the interdental space.

### Strengths

The strength of this study is the large number of sites investigated and its reflection of a close-to-real-life-picture of the utilization of IDBs. To the best of our knowledge, the following sentence, which is regularly encountered in the literature, “*The method of choice for interdental cleaning when brush space permits is IDB*” [6, 16], must be questioned. Screening of the accessibility of the interdental space should be a part of the routine examination in all patients. Its goal is to identify the distribution of IDB accessibility, site by site, and to choose the largest size, which passes between the teeth without causing discomfort [13]. An interdental brush, which is sized correctly for each interdental space, is easy to handle and will be atraumatic to the papillae [1].

### Limitations

There are no reports preceding this study on IDB rates specifically among the adult population. No gold standard tool in the literature for detecting interdental diameter currently exists; the development and validation of new tools that have the promise of high sensitivity and specificity to detect ID are critical, including a consensus on interpreting serial studies.

Periodontal biotype was not considered. If many factors have been correlated to the interdental papilla appearance, such as periodontal biotype or tooth morphology, interest has mainly been focused upon the dimensions that define the interdental area, which is the distance between the contact point and bone crest, as interradicular distances at different levels. Moreover, no scaling has been made before the screening, and calculus can only be removed by periodontal instrumentation. Our cross-sectional study could minimally impact the data by undervaluing the prevalence of the interdental diameter.

## Conclusions

Our previous results have indicated the need to introduce interdental brushing in young adults. Clinicians may find this novel format of data representation for a range of responses helpful in reaching decisions to manage biofilm and gingivitis for all patients. Interdental cleaning, similarly to toothbrushing, should become an established part of daily oral hygiene for the reduction of interproximal plaque, the control of gingivitis and improvement of patient motivation. In the context of the recent shift of biomedical science toward increased data sharing, it is important that the results from this study are disclosed to the scientific community, thereby contributing to evidence-based research in dentistry.

## Supporting Information

S1 Dataset Dataset. (XLS)
