# Correction: Access to Interdental Brushing in Periodontal Healthy Young Adults: A Cross-Sectional Study

**DOI:** 10.1371/journal.pone.0158252

**Published:** 2016-06-21

**Authors:** The PLOS ONE staff

The values in Tables 2 and 4 are incorrectly duplicated. In Table 2, lines 1–15 are the same as lines 16–31. Lines 16–31 feature the correct formatting. In Table 4, lines 1–11 are the same as lines 12–22. Lines 12–22 feature the correct formatting. Please see the corrected Tables 2 and 4 here. The publisher apologizes for the error.

**Table 2:** Distribution (mean ± sd) of the number of interproximal sites according to the diameter of the interproximal brush in 99 adults.

		IDB diameter (mm.)				
	n	0.6	0.7	0.8	0.9	1.1
**All**	99	8.78 ± 5.18	10.83 ± 4.56	3.39 ± 3.47	0.83 ± 1.42	0.49 ± 1.66
**Sex**						
Male	55	8.27 ± 5.07	10.80 ± 4.50	3.74 ± 3.52	0.98 ± 1.51	0.60 ± 2.01
Female	44	9.41 ± 5.31	10.86 ± 4.68	2.95 ± 3.38	0.64 ± 1.30	0.36 ± 1.06
p-value		0.280	0.944	0.260	0.229	0.510
**Smoker**						
No	75	9.03 ± 5.29	10.83 ± 4.35	3.11 ± 3.15	0.79 ± 1.33	0.37 ± 0.98
Yes	24	8.00 ± 4.86	10.83 ± 5.26	4.29 ± 4.27	0.96 ± 1.71	0.87 ± 2.89
p-value		0.594	0.991	0.142	0.615	0.195
**Subject's Baseline Bleeding**						
Low (< 30% bleeding sites)	39	7.85 ± 4.74	10.74 ± 4.87	4.18 ± 3.82	1.10 ± 1.82	0.67 ± 1.32
High (≥ 30% bleeding sites)	60	9.38 ± 5.40	10.88 ± 4.39	2.88 ± 3.14	0.65 ± 1.07	0.38 ± 1.84
p-value		0.147	0.877	0.065	0.118	0.587

http://dx.doi.org/10.1371/journal.pone.0155467.t002

**Table 4:** Distribution of interproximal sites (n = 2408^a^) from 99 adults according to adult’s variables.

		Brush diameter (mm.) (%↔)					
	n	0.6	0.7	0.8	0.9	1.1	p-value^b^
**Sex**							0.161
Female	1066	38.8	44.8	12.2	2.6	1.5	
Male	1342	33.9	44.3	15.4	4.0	2.5	
**Smoker**							0.205
Yes	599	32.1	43.4	17.2	3.8	3.5	
No	1809	37.4	44.9	12.9	3.3	1.5	
**Patient's periodontal risk**							0.061
Low (< 30% bleeding sites)	957	32.0	43.8	17.0	4.5	2.7	
High (≥ 30% bleeding sites)	1451	38.8	45.0	11.9	2.7	1.6	

^a^: Interproximal sites with enough space to introduce the IDB (interdental brush).

^b^: DESCRIPT procedure in SUDAAN 7.0 used to adjust for clustering (multiple sites within the patient).

http://dx.doi.org/10.1371/journal.pone.0155467.t004

## References

[1] Carrouel F, Llodra JC, Viennot S, Santamaria J, Bravo M, Bourgeois D (2016) Access to Interdental Brushing in Periodontal Healthy Young Adults: A Cross-Sectional Study. PLoS ONE 11(5): e0155467 doi: 10.1371/journal.pone.0155467 27192409 10.1371/journal.pone.0155467 PMC4871464

